# Hepatopulmonary Syndrome in a Patient With Autoimmune Hepatitis Without Liver Cirrhosis: A Case Report

**DOI:** 10.7759/cureus.64375

**Published:** 2024-07-11

**Authors:** Kyriakos Tarantinos, Vasiliki E Georgakopoulou, Aikaterini Nella, Dimitrios Mytas, Emmanouil Kastanakis

**Affiliations:** 1 First Department of Respiratory Medicine, Sismanogleio General Hospital, Athens, GRC; 2 Department of Pathophysiology/Pulmonology, Laiko General Hospital, Athens, GRC; 3 Second Department of Respiratory Medicine, Sismanogleio General Hospital, Athens, GRC; 4 Department of Cardiology, Sismanogleio General Hospital, Athens, GRC

**Keywords:** orthodeoxia, contrast-enhanced echocardiography, dlco, autoimmune hepatitis, hepatopulmonary syndrome

## Abstract

Intrapulmonary vasodilation leads to impaired arterial oxygenation, a hallmark of hepatopulmonary syndrome (HPS), a common pulmonary complication in end-stage liver disease. We present a case of HPS primarily diagnosed due to orthodeoxia in a 62-year-old ex-smoker with autoimmune hepatitis, under immunosuppressive treatment, but without liver cirrhosis. The patient reported dyspnea at rest that improved when supine. A recent chest CT scan showed no pulmonary embolism but indicated small nodules, bronchiectasis, and emphysema lesions. Functional breath monitoring revealed a low diffusing capacity for carbon monoxide (48% predicted). Blood gas analysis showed an increased alveolar-arterial difference, and contrast-enhanced echocardiography confirmed HPS with bubbles in the left heart chambers after the fourth cardiac cycle. Lung perfusion scintigraphy was negative for thromboembolic disease, but kidney imaging reinforced the HPS diagnosis. Our case is, to the best of our knowledge, the first presentation of HPS in a patient with autoimmune hepatitis without evidence of liver cirrhosis. This case highlights a rare instance of HPS in a patient with autoimmune hepatitis without liver cirrhosis, where orthodeoxia was the first clinical manifestation.

## Introduction

Hepatopulmonary syndrome (HPS) is a complex pulmonary vascular disorder characterized by the triad of liver disease, intrapulmonary vascular dilatations, and arterial hypoxemia [1]. It is a well-documented complication of end-stage liver disease, particularly cirrhosis, with a reported prevalence ranging from 4% to 47% in affected individuals [2]. However, HPS can also manifest in patients with acute or chronic hepatitis, portal hypertension without cirrhosis, and other hepatobiliary disorders, albeit less frequently [3].

While the pathogenesis of HPS remains incompletely understood, its hallmark feature is impaired arterial oxygenation due to abnormal pulmonary vascular dilatations, leading to ventilation-perfusion mismatch and hypoxemia [4]. Orthodeoxia, defined as a decrease in arterial oxygen saturation upon assuming an upright posture, is a distinctive clinical manifestation of HPS and underscores the complexity of its diagnosis [5].

We present a rare case of HPS with orthodeoxia as the initial clinical presentation in a patient with autoimmune hepatitis but without evidence of liver cirrhosis. This case challenges the traditional paradigm of HPS primarily occurring in the context of advanced liver disease and highlights the importance of considering this diagnosis in patients with hepatobiliary disorders beyond cirrhosis.

Through this case, we aim to raise awareness of the diverse clinical manifestations and diagnostic challenges associated with HPS, particularly in patients with autoimmune hepatitis and other non-cirrhotic liver conditions.

## Case presentation

A 62-year-old male ex-smoker with a documented history of autoimmune hepatitis, currently under immunosuppressive therapy, presented with complaints of dyspnea at rest that notably improved in the supine position. The patient's medical history was significant for the absence of liver cirrhosis, coronary artery disease, or pulmonary embolism over the past year, despite a dyspnea of unexplained etiology. The absence of liver cirrhosis was confirmed through a combination of clinical evaluation, laboratory tests (including liver function tests), and imaging studies (such as abdominal ultrasound and transient elastography), which showed no signs of cirrhosis.

**Figure 1:**
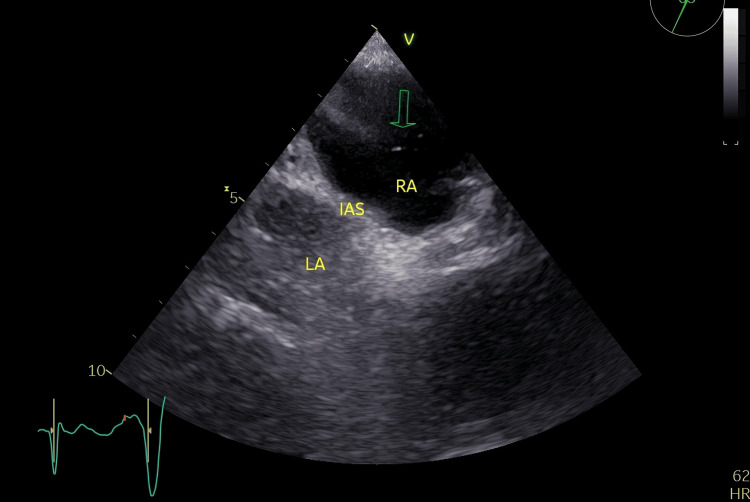
Contrast-enhanced echocardiography Presence of bubbles in the left heart chambers after the fourth cardiac cycle (arrow)

The patient had previously sought medical attention at a different hospital for similar symptoms a few days prior, where a chest CT scan following a pulmonary embolism protocol was performed. The scan was negative for pulmonary embolism but revealed several nodules measuring 2-5 mm, bronchiectasis, and emphysematous changes. Further pulmonary function testing indicated a reduced diffusing capacity for carbon monoxide (DLCO), measured at 48% of the predicted value. Arterial blood gas analysis highlighted an increased alveolar-arterial oxygen gradient (A-a gradient), with values of 57 mmHg in the supine position and 64 mmHg when upright. The presence of orthodeoxia coupled with the low DLCO raised clinical suspicion for HPS. To confirm the diagnosis, contrast-enhanced echocardiography was conducted. This imaging modality revealed the appearance of microbubbles in the left heart chambers starting from the fourth cardiac cycle, indicative of intrapulmonary vascular dilatations (Figure 1). 

**Figure 2:**
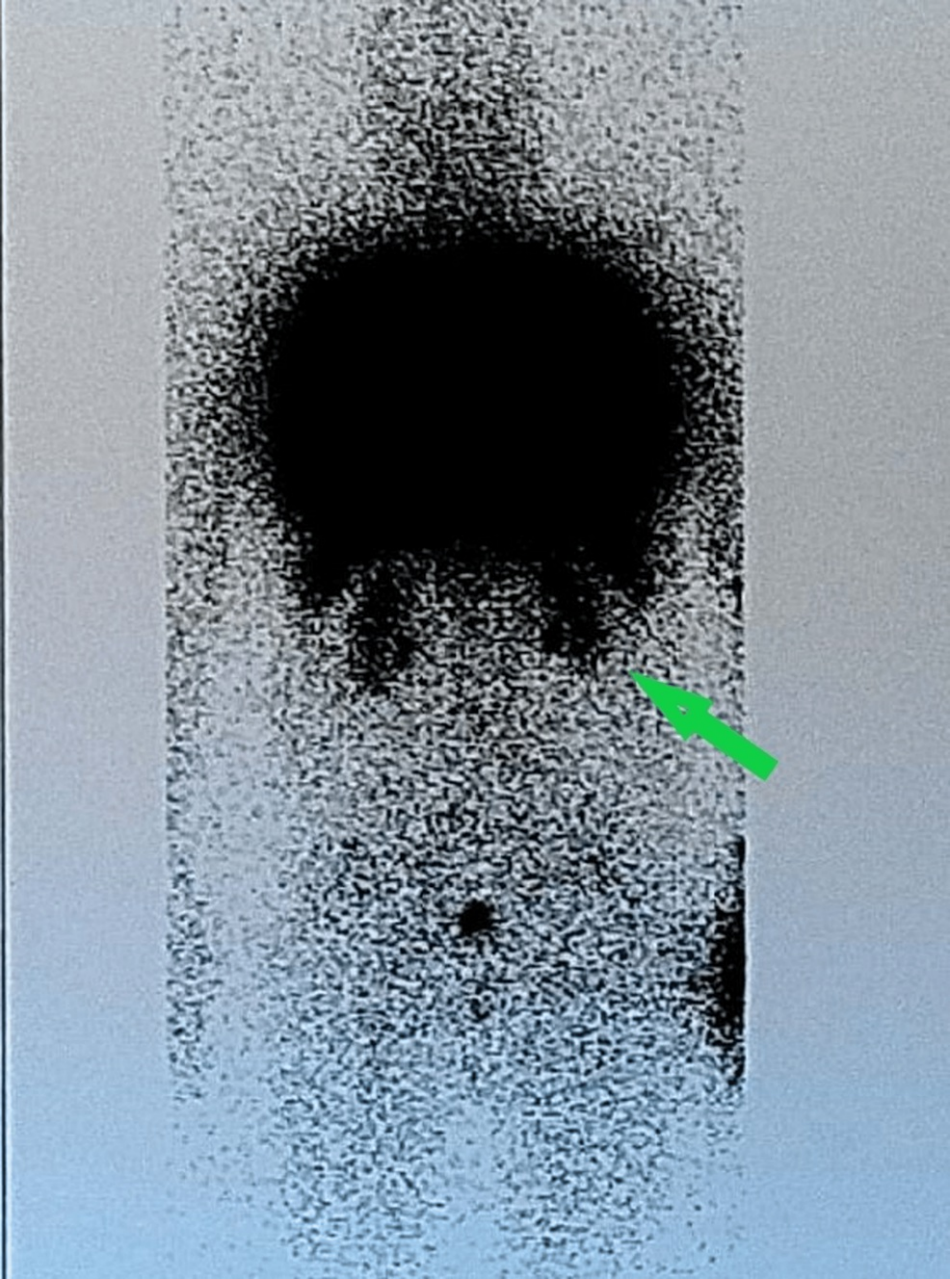
Scintigraphy of lung perfusion Imaging of the kidneys which under normal conditions are not shown (arrow).

These findings corroborated the diagnosis of HPS. Additionally, lung perfusion scintigraphy was performed to exclude thromboembolic disease, which yielded negative results. However, concurrent renal imaging during the scintigraphy further supported the diagnosis of HPS by demonstrating characteristic features consistent with the syndrome (Figure 2).

The patient was advised to continue his current immunosuppressive therapy and was referred for supplemental oxygen therapy to manage hypoxemia. He was also evaluated for potential liver transplantation, a definitive treatment for HPS. With supplemental oxygen, the patient's symptoms were expected to improve. If eligible for liver transplantation, this could potentially resolve the pulmonary symptoms and improve overall prognosis.

The patient reported improved dyspnea with supplemental oxygen therapy. His condition was monitored regularly, and he was placed on the liver transplant list. Over the following months, his hypoxemia remained stable with oxygen therapy, and he awaited transplantation for a long-term solution.

## Discussion

Our case is, to the best of our knowledge, the first presentation of HPS in a patient with autoimmune hepatitis without evidence of liver cirrhosis. This presentation challenges the traditional paradigm of HPS primarily occurring in the context of advanced liver disease and highlights the importance of considering this diagnosis in patients with autoimmune hepatitis presenting with unexplained hypoxemia.

A review of the literature reveals a limited number of reported cases of HPS in patients with other chronic liver diseases without cirrhosis, emphasizing the rarity of this presentation [6-9]. However, documented cases of HPS in various non-cirrhotic liver diseases provide valuable insights into the clinical manifestations and management strategies in similar patient populations [6-9].

Johnson et al. investigated HPS in non-cirrhotic patients and presented a case of a 34-year-old male with autoimmune lymphoproliferative syndrome and nodular regenerative hyperplasia [6]. Despite the absence of cirrhosis, the patient developed chronic hypoxia, with echocardiography revealing right-to-left intrapulmonary shunting consistent with HPS. His condition necessitated continuous oxygen therapy and liver transplantation, which resolved his hypoxia within 25 days post-transplant. This case underscores that HPS can occur in non-cirrhotic conditions like nodular regenerative hyperplasia, highlighting the need for awareness and early detection of HPS in non-cirrhotic liver diseases.

Teuber et al. found that pulmonary dysfunction, including impaired gas exchange, is common in non-cirrhotic patients with chronic viral hepatitis, with HPS diagnosed in 1.1% of these patients [7]. This finding indicates that HPS can occur even in the absence of cirrhosis, portal hypertension, or acute liver failure.

Elhabashy et al. reported that HPS can occur in non-cirrhotic patients with chronic viral hepatitis [8]. Their study on 60 patients revealed pulmonary dysfunction in 12, with 11.67% exhibiting hypoxemia. Intrapulmonary shunting was observed in three of these patients, and two met the diagnostic criteria for HPS. These findings highlight the need for pulmonary function monitoring in patients with chronic viral hepatitis.

Maya et al. presented a case of a female patient with non-cirrhotic portal hypertension who developed HPS due to chronic thrombosis of the portal and mesenteric veins caused by primary thrombophilia [9]. This case illustrates that HPS can arise from non-cirrhotic portal hypertension.

Soulaidopoulos et al. reviewed that HPS can coexist with genetic disorders like alpha-1 antitrypsin deficiency, metabolic diseases such as Wilson’s disease, and vascular malformations like Abernathy malformation [10].

Diagnostic evaluation of HPS in patients with autoimmune hepatitis without cirrhosis requires a comprehensive approach, including imaging modalities such as contrast-enhanced ultrasonography and lung perfusion scintigraphy [10]. These tests are crucial in confirming the diagnosis of HPS and excluding alternative causes of hypoxemia [10]. Additionally, arterial blood gas analysis demonstrating an elevated alveolar-arterial oxygen difference supports the diagnosis of HPS in the appropriate clinical context. Liver transplantation remains the only definitive treatment for HPS, significantly improving hypoxemia [10].

## Conclusions

In conclusion, while rare, HPS can indeed manifest in patients with autoimmune hepatitis without liver cirrhosis, with orthodeoxia serving as a significant clinical indicator. This case highlights the critical need for a thorough and systematic evaluation in patients with hepatobiliary disorders who present with unexplained hypoxemia. It is essential for clinicians to consider HPS in the differential diagnosis, even in the absence of cirrhosis, particularly when orthodeoxia is observed. The comprehensive diagnostic approach should include imaging modalities such as contrast-enhanced echocardiography and lung perfusion scintigraphy, alongside arterial blood gas analysis. These tools are vital for confirming HPS and ruling out other potential causes of hypoxemia. Early identification and intervention are pivotal in managing HPS effectively, as timely and appropriate treatment, including the possibility of liver transplantation, can significantly improve patient outcomes and prevent further disease progression. This case reinforces the importance of maintaining a high index of suspicion for HPS in similar clinical scenarios to optimize care and enhance prognostic prospects.
